# It’s All in the PAN: Crosstalk, Plasticity, Redundancies, Switches, and Interconnectedness Encompassed by PANoptosis Underlying the Totality of Cell Death-Associated Biological Effects

**DOI:** 10.3390/cells11091495

**Published:** 2022-04-29

**Authors:** Jessica M. Gullett, Rebecca E. Tweedell, Thirumala-Devi Kanneganti

**Affiliations:** Department of Immunology, St. Jude Children’s Research Hospital, Memphis, TN 38105, USA; jessica.gullett@stjude.org (J.M.G.); rebecca.tweedell@stjude.org (R.E.T.)

**Keywords:** PANoptosis, PANoptosome, pyroptosis, apoptosis, necroptosis, inflammatory cell death, inflammasome, inflammation, innate immunity, infection, NLR, caspase, IRF1, ZBP1, RIPK1, RIPK3, MLKL, NLRP3, AIM2, Pyrin, caspase-1, ASC, caspase-8, caspase-3, caspase-7, crosstalk, plasticity, redundancy

## Abstract

The innate immune system provides the first line of defense against cellular perturbations. Innate immune activation elicits inflammatory programmed cell death in response to microbial infections or alterations in cellular homeostasis. Among the most well-characterized programmed cell death pathways are pyroptosis, apoptosis, and necroptosis. While these pathways have historically been defined as segregated and independent processes, mounting evidence shows significant crosstalk among them. These molecular interactions have been described as ‘crosstalk’, ‘plasticity’, ‘redundancies’, ‘molecular switches’, and more. Here, we discuss the key components of cell death pathways and note several examples of crosstalk. We then explain how the diverse descriptions of crosstalk throughout the literature can be interpreted through the lens of an integrated inflammatory cell death concept, PANoptosis. The totality of biological effects in PANoptosis cannot be individually accounted for by pyroptosis, apoptosis, or necroptosis alone. We also discuss PANoptosomes, which are multifaceted macromolecular complexes that regulate PANoptosis. We consider the evidence for PANoptosis, which has been mechanistically characterized during influenza A virus, herpes simplex virus 1, *Francisella novicida*, and *Yersinia* infections, as well as in response to altered cellular homeostasis, in inflammatory diseases, and in cancers. We further discuss the role of IRF1 as an upstream regulator of PANoptosis and conclude by reexamining historical studies which lend credence to the PANoptosis concept. Cell death has been shown to play a critical role in infections, inflammatory diseases, neurodegenerative diseases, cancers, and more; therefore, having a holistic understanding of cell death is important for identifying new therapeutic strategies.

## 1. Introduction

The innate immune system is the first line of defense against infection and cellular insults; innate immune receptors can recognize the molecular signatures of pathogens, called pathogen-associated molecular patterns (PAMPs), as well as components released by damaged cells, called damage-associated molecular patterns (DAMPs). The innate immune system activates genetically defined programmed cell death pathways in response to microbial infections or alterations in cellular homeostasis; among the most well characterized of these programmed cell death responses are pyroptosis, apoptosis, and necroptosis. Though canonically proposed as segregated cellular processes responding to individualized PAMPs and DAMPs, mounting evidence shows significant interactions between the components of pyroptosis, apoptosis, and necroptosis. Historically, the literature on cell death and innate immune signaling has used different terms to describe these interactions, such as ‘crosstalk’, ‘plasticity’, ‘redundancies’, and ‘molecular switches’. Consideration of the totality of biological effects from cell death in multiple studies has led to the conceptualization of PANoptosis [1,2,3,4,5,6,7,8,9,10,11,12,13,14,15,16,17,18,19,20], an inflammatory cell death pathway that integrates components from other cell death pathways. PANoptosis is implicated in driving innate immune responses and inflammation and cannot be individually accounted for by pyroptosis, apoptosis, or necroptosis alone. PANoptosis is regulated by PANoptosomes, multifaceted macromolecular complexes. Here, we review the key components of programmed cell death pathways and highlight the plasticity among pyroptosis, apoptosis, and necroptosis. We then discuss the conceptualization of PANoptosis, which continues to evolve over time based on data, and examine the current evidence supporting this concept. 

### Key Components in Inflammatory Programmed Cell Death Pathways 

Among the most comprehensively studied cell death processes to date are pyroptosis, apoptosis, and necroptosis [21,22]. Each occurs in response to cellular insults, but they differ in terms of their molecular machinery. Pyroptosis is a lytic form of proinflammatory cell death that was originally described as a caspase-1-mediated death [23]. Pyroptotic cell death typically involves the formation of the inflammasome, a supramolecular platform that is composed of a sensor, adaptor protein ASC, and caspase-1 [24]. The five most well-known inflammasomes, which are named after their corresponding sensor based on the genetic characterization of sensors and triggers, are the NLR family inflammasomes, NLRP1 [24], NLRP3 [25,26,27], and NAIP/NLRC4 [28,29,30], as well as those formed by other sensors containing pyrin domains, such as Pyrin [31] and AIM2 [32,33]. Sensor activation polymerizes the adaptor protein ASC into prion-like structures referred to as ASC specks [34,35,36], which recruit caspase-1 to allow its autoproteolysis and activation [24,37]. Activated caspase-1 cleaves inflammatory cytokines IL-1β and IL-18 [38] as well as the pore-forming molecule gasdermin D (GSDMD) [39]. The GSDMD-mediated pores allow the release of the inflammatory cytokines [40,41,42,43,44] along with other inflammatory molecules such as HMGB1, which serve as DAMPs and further propagate an innate immune inflammatory response. GSDMD is also activated by caspase-11 (mice) and caspase-4/5 (humans) in the process of non-canonical inflammasome activation [39,40,41,44,45]. In addition to requiring cleavage for activation, GSDMD is also regulated at the transcriptional level by IFN regulatory factor 2 (IRF2), with a compensatory role for IRF1, in murine bone marrow-derived macrophages (BMDMs) [46]; in human cells IRF2 does not regulate GSDMD expression but does regulate caspase-4-mediated cell death, and IRF1 acts cooperatively in this process in response to IFN-γ [47]. 

Apoptosis is a form of programmed cell death originally described as a ‘mechanism of controlled cell deletion’ characterized by its distinct morphological membrane blebbing and subsequent cell shrinkage [48]. It proceeds through either an extrinsic or intrinsic pathway, though both result in the activation of the same executioner caspases. The intrinsic pathway forms an APAF1-mediated apoptosome in response to homeostatic disruptions, such as DNA damage or loss of mitochondrial stability [49]. This multiprotein complex includes APAF1, cytochrome c, and the initiator caspase caspase-9 which, upon cleavage, activates the downstream effector/executioner caspases, caspase-3 and -7 [50,51]. Extrinsic apoptosis occurs after ligand binding to death receptors, such as Fas and TNF-α receptor (TNFR), on the cell surface; downstream of death receptor binding, FADD translocates to the receptor, which recruits caspase-8. Caspase-8 is the key extrinsic apoptotic initiator caspase which cleaves downstream caspases, caspase-3 and -7, to execute cell death [52,53]. Caspase-8 can also induce activation of intrinsic apoptosis by activating the proapoptotic molecule Bid [54,55,56], which translocates to the mitochondria to facilitate pore formation by BAX/BAK and induce mitochondrial outer membrane permeabilization (MOMP) and apoptosome formation [57,58].

Necroptosis, another lytic form of cell death, occurs in response to caspase-8 inhibition and is RIPK3- and MLKL-dependent [22,59,60,61]. The apoptotic caspase-8 typically blocks necroptosis by cleaving RIPK3, CYLD, and RIPK1 [62,63,64]. Necroptosis can be initiated in response to the activation of toll-like receptors (TLRs), death receptors, or through interferon (IFN) signaling [65]. A well-characterized necroptosis response is induced by TNF-α. Its binding to TNFR induces signaling that activates RIPK1 to become phosphorylated and, along with TRADD, FADD, and caspase-8, form complex II [66]. When caspase-8 is inhibited, RIPK1 interacts with RIPK3 to form a cell death-inducing necrosome. The RIPK1-RIPK3 complex promotes phosphorylation of MLKL, causing its oligomerization. The MLKL multimer then translocates to the plasma membrane, where it interacts with phospholipids and forms pores [67,68]. In addition to MLKL phosphorylation by RIPK3, MLKL activity is also regulated by other post-translational modifications, such as ubiquitylation, which is necessary for higher-order oligomerization [69]. 

Within each of these cell death pathways, there are several regulators and auxiliary components. For example, additional inflammasome components have been identified that are involved in its regulation and activation, such as NEK7 [70,71,72,73] and DDX3X [74], as well as transcription factors such as IRF1 [75], IRF2 [46,47], and IRF8 [76]. Additionally, NINJ1 has been identified as a critical component for plasma membrane rupture [77]. There are also variations in the signaling cascades that exist within each programmed cell death pathway, making the complexity of these cellular processes, including regulatory components, cell- and trigger-specific responses, and time-dependent responses, limitless. Additional components and layers of complexity have been extensively reviewed elsewhere [22,78,79]. 

## 2. Evidence of Crosstalk at the Molecular Level

Understanding the activation and execution of inflammatory cell death pathways has been an active area of research, particularly given the clinical relevance of cell death pathways in infections, inflammatory diseases, cancers, and beyond [2,4,5,7,8,9,11,12,13,16,18,19,20]. As a result of these studies, several examples of crosstalk and flexibility have been identified between the molecular components of programmed cell death pathways. Here, we will limit our discussion to genetically defined examples over time. 

At their core, apoptosis and necroptosis are intricately molecularly linked, given that TNF-induced caspase-8 activation drives apoptosis while inhibition of caspase-8 during this process drives necroptosis [80]. The rescue of caspase-8-deficient embryos by the loss of RIPK3 or MLKL has long been documented [81,82,83,84], and enzymatically active caspase-8 is critical in the regulation and balance of apoptosis and necroptosis [85,86]. 

Beyond the intrinsic connection between apoptosis and necroptosis, caspase-1, an essential component of inflammasomes, cleaves apoptosis-associated caspase-7 during *Salmonella* infection (NLRC4 inflammasome trigger) as well as in response to LPS + ATP stimulation (NLRP3 inflammasome trigger) [17]. The pyroptotic caspase-1 also cleaves apoptotic PARP1 in response to inflammasome-activating triggers [6], and loss of caspase-1 during *Salmonella* infection leads to activation of apoptotic proteins instead [87]. In addition, cells lacking pyroptotic caspase-1 and caspase-11 have reduced mitochondrial damage in response to inflammasome-activating triggers such as the NLRP3-activating LPS + ATP treatment or AIM2-activating dsDNA transfection [88], suggesting additional crosstalk between inflammasomes and apoptotic processes. Reciprocally, the apoptotic caspase-8 serves as a regulatory component of pyroptotic inflammasomes [19]. Fluorescence microscopy has shown the colocalization of caspase-8 and ASC in both pyroptosis-deficient and pyroptosis-sufficient cells in response to infections [12,89,90,91]. Additionally, caspase-8, along with FADD, is required to both prime and activate canonical (ligand-induced) and noncanonical (*E. coli*- or *Citrobacter rodentium*-induced) NLRP3 inflammasomes [19]. Caspase-8 can be recruited during NLRC4 and NLRP1b inflammasome formation [91,92,93] and at ASC specks involving multiple inflammasome sensors, such as NLRP3 and NLRC4 or AIM2 and Pyrin [12,94]; FADD can also be recruited to these ASC specks in response to FlaTox, a combination of the bacterial PAMPs *Bacillus anthracis* protective antigen and the N-terminus of lethal factor fused to *Legionella pneumophila* flagellin [93]. However, caspase-8 is not required for *Salmonella*-induced cell death at 2, 6, and 24 h post-infection using an MOI of 1 or 10 [91], showcasing the variability of the roles of caspase-8 within the programmed cell death response. 

Crosstalk has also been identified between cell death molecules by studying the totality of biological effects in disease processes. For example, inflammatory bone disease in mice carrying the *Pstpip2*^cmo^ mutation persists despite deletion of caspase-1 or combined deletion of caspase-8/RIPK3 (deletion of caspase-8 alone is embryonically lethal [84]); the inflammation is only rescued by the combined deletion of NLRP3 or caspase-1 with caspase-8/RIPK3 [16,18], highlighting the functional redundancies of pyroptotic molecules NLRP3 and caspase-1 with the apoptosis-necroptosis modulator caspase-8. In the context of infection, influenza A virus (IAV) induces activation of pyroptotic, apoptotic, and necroptotic proteins, and loss of RIPK3 protects against much of the cell death, but combined deletion of caspase-8 and RIPK3 is necessary to further reduce cell death [9], providing additional mechanistic evidence of overlaps in the functions of molecules involved in cell death activation.

Beyond caspase-8 and RIPK3, the necroptotic molecule MLKL has also been implicated in crosstalk between cell death pathways. For example, ASC oligomerization to induce NLRP3 inflammasome activation can occur in response to treatment with TLR3 ligands and zVAD, but the ASC oligomerization is blocked in MLKL-deficient cells [95]. As oligomerized MLKL forms pores in the plasma membrane, a cascade of cellular consequences begins, including the efflux of potassium ions. This necroptosis-induced ionic efflux has been shown to activate the NLRP3 inflammasome [96,97]. Together, these data show how necroptosis and inflammasomes (pyroptosis) are interconnected. 

Given the recently identified role of gasdermins in cell death, it has also been found that gasdermins mediate crosstalk between cell death pathways. GSDMD was initially identified as an executioner of pyroptotic cell death in response to caspase-1, caspase-4/-5 (human) or caspase-11 (mouse) cleavage [39,45]. Caspase-8 can also cleave GSDMD to activate pore formation and cell death during *Yersinia* infection [3,98,99,100]. Further studies have found that GSDMD can also be processed by the apoptosis-inducing caspase-3 in such a manner that renders GSDMD inactive, suppressing pyroptosis [101]. However, inflammasome and GSDMD activation in response to Shiga toxin 2 and LPS are also associated with increased mitochondrial ROS [102], and GSDMD can form pores in the mitochondrial membrane to release canonically proapoptotic molecules and activate caspase-3 in a BAK/BAX-independent manner [103,104]. Other members of the gasdermin family are also increasingly implicated in cell death crosstalk. Microarray and subsequent pathway analysis of inner ear samples from day-0 postnatal mice showed that the gene set involved in apoptosis is downregulated in mice lacking *Gsdme* as compared with wild-type controls [105]. Furthermore, GSDME can be cleaved by caspase-3, an apoptotic cell death effector, and can induce pyroptotic death [106,107]. In THP-1 cells lacking GSDMD, GSDME allows the release of IL-1β in response to nigericin, Val-boroPro, or *Salmonella* infection, though limited cell death was observed with endogenous GSDME expression levels in these cells [108]. In murine cells, NLRP3 inflammasome activation in GSDMD-deficient cells results in IL-1β and IL-18 release through caspase-8/-3 and GSDME activation [109]. GSDME serves in a feed-forward loop to promote caspase-3 activation by forming pores in the mitochondrial membrane and inducing the release of cytochrome c in response to traditional intrinsic and extrinsic apoptotic stimuli; overexpression studies have shown similar results with GSDMA [103]. Beyond these connections, in cells lacking pyroptosis via GSDMD-deficiency, caspase-1 can cleave caspase-3 and Bid to promote apoptotic cell death in response to inflammasome triggers such as LPS priming and poly(dA:dT) transfection, or during *Salmonella* infection [110,111]. Furthermore, the APAF1-apoptosome has been shown to interact with caspase-11 when cells are challenged with bile acid; the result is caspase-3 cleavage and the execution of pyroptotic death in a GSDME-mediated process [112]. Other pore-forming molecules may also be involved in this crosstalk, as pannexin-1 activation downstream of caspase-8 or -9 activation leads to NLRP3 inflammasome formation in a GSDMD- and GSDME-independent process [113]. 

## 3. Prototypical Examples of PANoptosis

**Figure 1:**
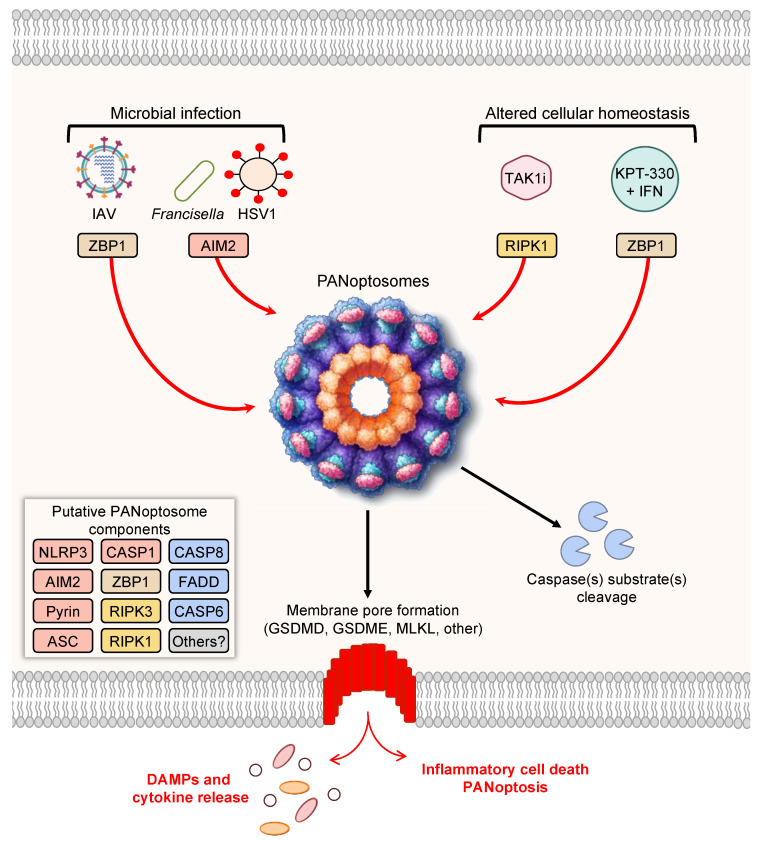
PANoptosis and PANoptosome formation. Upon exposure to cellular insults, such as microbial infection or altered cellular homeostasis, sensors can detect the perturbation and activate PANoptosis. Prototypical examples of PANoptosis are depicted here. Sensor activation can lead to the formation of a multiprotein complex, the PANoptosome. PANoptosomes have the potential to bring together diverse components from previously segregated cell death pathways. These may be dynamic complexes, and their protein composition may vary in trigger- and time-dependent manners. Potential PANoptosome components putatively include inflammasome sensors, such as nucleotide-binding oligomerization domain-like receptor family pyrin domain-containing 3 (NLRP3), absent in melanoma 2 (AIM2), Pyrin, Z-DNA-binding protein 1 (ZBP1), or others; apoptosis-associated speck-like protein containing a caspase activation and recruitment domain (ASC); caspase-1 (CASP1); receptor-interacting serine/threonine protein kinase 3 (RIPK3); RIPK1; caspase-8 (CASP8); Fas-associated protein with death domain (FADD); and/or caspase-6 (CASP6). PANoptosis involves membrane pore formation for the execution of cell death to release cytokines, such as IL-1β and IL-18, and DAMPs. Figure created with https://biorender.com/ (accessed on 17 March 2022).

The depth and breadth of literature encompassing innate immune signaling and programmed cell death is impressive. Repeatedly, the literature acknowledges instances of crosstalk between components of pyroptosis, apoptosis, and necroptosis. This crosstalk occurs in context-dependent manners and is sometimes referred to as ‘plasticity’ or ‘redundancy’, with cell death components often labeled as ‘molecular switches’. The overwhelming amount of evidence for the interconnectedness between cell death pathways has led to the conceptualization of PANoptosis as an inflammatory cell death pathway. The totality of biological effects in PANoptosis cannot be individually accounted for by pyroptosis, apoptosis, or necroptosis alone [2,3,4,5,6,7,8,9,10,11,12,13,14,15,16,17,18,19,20]. PANoptosis has been increasingly implicated in infectious and inflammatory diseases as well as in cancers and cancer therapies [2,3,4,5,7,8,9,10,11,12,13,14,15,16,18,20,114,115,116,117,118]. Here, we will focus on the most mechanistically well-characterized examples of PANoptosis and PANoptosomes [3,7,9,10,11,12,20] (Figure 1). 

As the conceptualization of PANoptosis implies, PANoptosis involves the activation of several molecules previously characterized as mediators of independent cell death pathways. For instance, Z-DNA-binding protein 1 (ZBP1) was previously known to induce necrosis in response to a mutant form of MCMV expressing a tetra-alanine RHIM substitution in vIRA (M45*mut*RHIM) [119] and was shown to interact with the necroptotic molecules RIPK1 and RIPK3 [120], but more recent evidence has shown that ZBP1 acts as a cytosolic innate immune sensor for endogenous nucleic acids or during IAV infection to induce activation of the NLRP3 inflammasome, caspase-1, caspase-8, caspase-3, caspase-7 [7,9,11], and MLKL [7,11,121]. Molecularly, ZBP1 mediates the formation of a multiprotein ZBP1-PANoptosome complex, containing ZBP1, RIPK3, RIPK1, caspase-8, caspase-6, ASC, and NLRP3 [10,11]. To date, this complex has been characterized by immunoprecipitation [7,10,11], and immunofluorescence has also shown colocalization of caspase-8 and RIPK3 with ASC specks in individual cells during IAV infection [122]. Further studies, including biochemical analyses and cryo-EM evaluation, are needed to fully understand how components come together in individual cells. The ZBP1-PANoptosome complex can also be implicated in tumorigenesis, where ADAR1 acts as a negative regulator to prevent the interaction between ZBP1 and RIPK3 and promote tumorigenesis. Limiting the interaction between ADAR1 and ZBP1 by sequestering ADAR1 in the nucleus, through treatment with nuclear transport inhibitors (KPT-330) in conjunction with IFN, potentiates PANoptosis and limits tumorigenesis [7]. 

Additionally, the inflammasome sensor AIM2 also initiates the formation of PANoptosome complexes during herpes simplex virus 1 (HSV1) and *Francisella novicida* infections. This PANoptosome, termed the AIM2-PANoptosome, contains AIM2, ZBP1, Pyrin, ASC, caspase-1, caspase-8, RIPK3, RIPK1, and FADD [12]. PANoptosomes have also been identified by immunoprecipitation during *Yersinia* infection, where RIPK1, RIPK3, caspase-8, FADD, ASC, and NLRP3 can be co-immunoprecipitated [3]. In the context of *Yersinia* infection, RIPK1 is necessary for activation of caspase-1, GSDMD, caspase-8, caspase-3, and caspase-7, but it negatively regulates the activation of MLKL, highlighting the multifaceted modulation of cell death effectors that can occur within PANoptosis [3]. PANoptosis has also been observed in response to TAK1 inhibition in macrophages, which can occur as a result of *Yersinia* infection due to its effector YopJ or in response to genetic mutations or treatment with TAK1 inhibitors [5,20,98,100]. In the case of TAK1-deficient macrophages, spontaneous PANoptosis occurs and is characterized by the activation of the NLRP3 inflammasome, caspase-1, caspase-3, caspase-8, and MLKL [5,20]; stimulation with LPS in TAK1-deficient macrophages induces colocalization of RIPK1, ASC, and caspase-8 in a RIPK1 kinase-independent manner [20]. 

## 4. PANoptosis Regulation via IRF1

As with other cell death pathways, PANoptosis must be tightly regulated to control the execution of cell death. IRF1, a molecule long recognized for its roles in regulating cell death [123,124], is a key upstream regulator of PANoptosis. In the absence of IRF1 during IAV infection, ZBP1 protein expression, along with NLRP3 inflammasome, caspase-1, caspase-8, caspase-3, and MLKL activation, are all reduced [75]. In the context of colorectal tumorigenesis, IRF1 facilitates the activation of PANoptosis to limit tumorigenesis [8]. PANoptosis has also been observed to be driven by IRF1 in response to the combination of TNF and IFN-γ. TNF and IFN-γ release can occur physiologically during cytokine storm syndromes, including during SARS-CoV-2 infection [2], and together they induce PANoptosis through the JAK/IRF1 signaling axis [2,4]; this observation has led to a mechanistic definition for cytokine storm as a life-threatening condition caused by excessive production of cytokines mediated by PANoptosis [125]. Additionally, the AIM2 inflammasome has also been shown to be regulated by IRF1 during *Francisella* infection [126], suggesting a possible regulatory role of IRF1 in PANoptosis mediated by the AIM2-PANoptosome, although this remains to be investigated. 

## 5. A Rose by Any Other Name

Given the extensive history characterizing innate immune signaling and cell death molecules, complexes, and processes, it is important to revisit previous studies to potentially connect key concepts. The recent expansion of studies on PANoptosis further combined with a fresh look at the full body of literature in the cell death field suggests that the concept of PANoptosis has been hiding in plain sight (Table 1 and Table 2). Many researchers have reported instances of cell death crosstalk, functional redundancy, or interconnectedness [127], all of which sets the foundation for PANoptosis. As one key example, a wealth of literature exists on the apoptotic caspase-8 and how its loss or impaired functionality modulates multiple programmed cell death pathways and their plasticity in development and disease [62,128,129,130,131,132]. Mice expressing a catalytically inactive version of caspase-8, i.e., *Casp8*^C362A/C362A^, are embryonically lethal [130]. Cells carrying *Casp8*^C362A/C362A^ form ASC specks, induce activation of the inflammatory caspase substrate GSDMD, and activate apoptotic caspases, caspase-3 and caspase-7, to mediate cell death. While embryonic lethality in mice carrying *Casp8*^C362A/C362A^ can be partially rescued by deleting pyroptotic caspase-1 or ASC, mice with combined deletions of caspase-1/-11/RIPK3 have the best survival outcome [130], suggesting that multiple cell death pathways are responsible for the embryonic lethality. Similar results are observed in mice carrying another enzymatically inactive caspase-8 mutation, *Casp8*^C362S/C362S^, where lethality is rescued by combined deletion of MLKL/ASC or MLKL/caspase-1 [128], suggesting that the apoptotic caspase-8 can play a scaffolding role, and multiple cell death effectors are involved in the cell death in mice carrying catalytically inactive caspase-8. On the one hand, mice carrying a non-cleavable version of caspase-8 (*Casp8^D^*^387*A*/D387A^ [*Casp8*^DA/DA^] or homozygous *Casp8*^D212A/D218A/D225A/D387A^ [*Casp8*^4DA/4DA^]) are viable and normal [62,129,132], while, on the other hand, *Casp8*^DA/DA^*Mlkl*^−/−^ and *Casp8*^DA/DA^*Ripk3*^−/−^ mice develop extensive inflammation. This inflammation can be rescued by deletion of one allele of FASL, FADD, or RIPK1 [129], implicating apoptotic and necroptotic molecules in this inflammation. Furthermore, cell type-specific deletions of caspase-8 have identified additional connections. Mice lacking intestinal epithelial cell (IEC) caspase-8 develop colitis and ileitis which can be rescued by deletion of MLKL. Colitis and ileitis also occur in mice lacking FADD in IECs, with cell death mediated by MLKL and caspase-8-dependent activation of GSDMD. Upstream, loss of ZBP1 is sufficient to prevent ileitis in both mice with caspase-8- and FADD-deficient IECs [131]. Collectively, these results align well with the PANoptosis concept, where there is a totality of biological effects in the phenotype that do not fit within pyroptosis, apoptosis, or necroptosis alone. 

Many other in vivo disease phenotypes can be assessed through the PANoptosis lens. For instance, the osteomyelitic disease observed in mice carrying a *Pstpip2*^cmo^ mutation is likely associated with PANoptosis. The bone disease in these mice is driven by aberrant production of IL-1β and is associated with inflammasome activation and cell death. Given the clear role of inflammasome-mediated cytokine release in the disease, it seemed likely that deletion of pyroptotic molecules, such as NLRP3 or caspase-1, would rescue the disease. However, this was not the case. Only mice lacking combinations of cell death molecules, including NLRP3/caspase-8/RIPK3 or caspase-1/-8/RIPK3, are protected from disease [16,18]. Similar results are observed in the *Sharpin*^cpdm^ mouse model, where mutations in SHARPIN, a linear ubiquitin chain assembly complex component critical for TNF signaling activation, result in skin inflammation. In these mice, deletion of NLRP3 or caspase-1 [142] or MLKL alone [143] delays, but does not prevent, the skin inflammation, while combined deletion of FADD/RIPK3 in epidermal cells rescues the inflammation [144]. Additionally, footpad inflammation in mice carrying the *Ptpn6*^spin^ mutation is not rescued by single deletions of caspase-1, NLRP3, RIPK3, MLKL, or the combined deletion of caspase-8/RIPK3 [147,148]. The inflammatory disease in these mice, which resembles neutrophilic dermatosis in humans, is mediated by the RIPK1/IL-1α signaling axis, but combined deletion of caspase-8/RIPK3/RIPK1 is needed to prevent the inflammation [148]. Furthermore, innate immunity and programmed cell death have been connected to neurodegenerative diseases such as Alzheimer’s disease (AD). Elevated levels of proinflammatory cytokines that can be released by cell death, such as TNF-α and IL-1β, are found in the brains and serum of people with AD and can cause neuroinflammation and AD-related pathologies [153]. The potential for PANoptosis is shown in studies with amyloid precursor protein/presenilin 1 (APP/PS1) transgenic mice carrying kinase-dead RIPK1 (*Ripk1*^D138N^). A deficiency in functional RIPK1 results in reduced disease and decreased proinflammatory IL-1β release [154], linking necroptotic and pyroptotic molecules in this model. Given the multifaceted nature of the cell death in many in vivo disease phenotypes, it is likely that many others could also be considered within the PANoptosis concept. 

Several infection models have also demonstrated cell death crosstalk and interconnectivity that can be considered within the PANoptosis concept. As discussed above, ZBP1-mediated cell death during IAV infection is the prototypical example of PANoptosis. In initial studies, IAV-induced cell death was often viewed as redundant or thought to concurrently activate multiple cell death pathways [75,121,155], but later work elucidated the molecular mechanisms of ZBP1-PANoptosome formation and established the complex [11]. ZBP1-mediated PANoptosis has also been implicated in tumorigenesis [7] and fungal infections with *Candida albicans* and *Aspergillus fumigatus* [14]. In response to other viruses, PANoptosis mediated through AIM2-PANoptosome formation has also been characterized during HSV1 infection [12]. Additionally, during hepatitis C virus infection, fluorescence microscopy has shown concurrent caspase-1 and caspase-3 activation [156], suggesting simultaneous activation of these molecules in individual cells, which could be explained by PANoptosis. 

Bacterial pathogens have also been found to induce cell death where the molecular phenotypes observed do not fall within pyroptosis, apoptosis, or necroptosis activation alone, suggesting PANoptosis is occurring. In response to FlaTox, mice lacking either the combination of caspase-1/RIPK3 or the combination of caspase-8/RIPK3 still experience pathology, while combined loss of caspase-1/-8/RIPK3 provides protection [151]. Similarly, co-deletion of ASC or caspase-8/RIPK3 with caspase-1/-11 phenocopies NLRC4 deletion during challenge with flagellin or infection with *Legionella* [92,151]. In *Salmonella* infection, ’flexible’ cell death has been observed, and it has been reported that components of different cell death pathways interact. *Salmonella*-induced NLRC4 inflammasome activation drives cell death to clear bacteria from the host, and mice deficient in RIPK3 and caspase-1 are moderately impaired in their ability to clear the bacteria, while bacterial clearance in mice deficient in RIPK3, caspase-1, and caspase-8 is significantly more impaired [151]. Subsequent studies have shown that cells deficient in caspase-1/-11 or caspase-8/RIPK3 still undergo cell death in response to the infection, while cells deficient in caspase-1/-11/-8/RIPK3 [10] or cells deficient in caspase-1/-11/-12/-8/RIPK3 [135] are protected from cell death. Infection with *Bacillus anthracis* also activates multiple cell death effectors, including caspase-1, -8, and -3. Deletion of RIPK3 is not sufficient to protect cells from death during this infection, while the combined deletion of caspase-8/RIPK3 does prevent cell death [157]. Additionally, *Pseudomonas aeruginosa* induces cell death characterized by activation of caspase-1, GSDMD, caspase-8, -3, -7, and MLKL. While deletion of the pyroptotic sensors NLRP3 and NLRC4 together significantly reduces cell death in response to *P. aeruginosa*, combined deletion of caspase-1/-11/-8/RIPK3 provides full protection from cell death [137]. As each of these phenotypes cannot be explained by activation of pyroptosis, apoptosis, or necroptosis alone, they may fit in the category of PANoptosis. 

Having PANoptosis as a component of the innate immune response is likely advantageous on the organismal level. Inhibiting cell death effectors is a commonly used pathogen defense strategy [158,159,160], and PANoptosis allows the cell to utilize multiple routes of cell death if one or more key cell death effectors is inhibited. Biologically, the redundancy displayed by PANoptosis ensures a fail-safe mechanism for cell death, thereby increasing the likelihood of organismal survival. During *Yersinia* infection, the bacteria secrete YopJ, a bacterial effector that inhibits TAK1-dependent inflammatory signaling [98,100]. However, in response to the inhibition of TAK1, host cells undergo cell death characterized by activation of the NLRP3 inflammasome, caspase-1, -3, -7, and MLKL [5]. Recently, *Yersinia* infection was found to activate PANoptosis, with infection inducing the formation of a PANoptosome containing RIPK1, RIPK3, caspase-8, ASC, FADD, and NLRP3 [3]. *Shigella flexneri* also uses its effector molecules, OspC1 to inhibit caspase-8 and OspD3 to inhibit RIPK1/RIPK3, in an attempt to evade the cell death crosstalk [161], which could give this bacterium a strategy to prevent PANoptosis.

## 6. Discussion and Future Directions

Experimental design often restricts how we test the innate immune response, limiting our ability to fully determine connections between molecular processes. The host response is likely more complex than we understand, but we can appreciate that the host must be able to recognize and respond to a variety of danger signals and cellular insults to execute the appropriate cellular response. It would likely be evolutionarily advantageous for a barrage of cellular insults to be neutralized using an integrated immune response, such as that stemming from PANoptosis. Therefore, it is important to be inclusive and consider the totality of biological effects in PANoptosis when studying cell death. These phenotypes may be missed when focusing on a single cell death pathway due to functional redundancies and overlaps between molecules. Additionally, the activation of molecular executioner signatures of pyroptosis, apoptosis, and necroptosis are not required simultaneously in an individual cell for a cell death process to fit within the PANoptosis concept. Furthermore, there are certainly conditions where pyroptosis, apoptosis, or necroptosis alone carries out the cell death, such as pyroptosis in wild-type cells exposed to LPS + ATP or necroptosis in wild-type cells exposed to TNF + zVAD. Only by studying the totality of the effects of programmed cell death in physiologically relevant models can we identify the important differences between these instances and PANoptosis. 

Multiple PANoptosome complexes have been associated with PANoptosis to date [3,7,9,10,11,12,20], and additional work is required to fully understand these complexes. Their molecular composition may be flexible and dynamic. In the case of other cell death-inducing complexes, such as the NLRP3 inflammasome, there are canonical and non-canonical iterations; the same may also be true for PANoptosomes. Canonical PANoptosomes may contain caspase-8, RIPK3, and inflammasome components, as has been observed during IAV, HSV1, *Francisella*, and *Yersinia* infections [3,10,11,12], while non-canonical PANoptosomes may contain caspase-8 and RIPK3 as major molecules, as is likely the case during TNF + IFN-γ stimulation [2,4]. More work is needed to continue to evaluate these complexes, the dynamics of their formation, and their regulation. Furthermore, in addition to the ZBP1- and AIM2-PANoptosome complexes that have been molecularly characterized based on their upstream sensors, it is likely that many other sensor-specific PANoptosomes exist and form in response to infections and conditions of altered cellular homeostasis that have not yet been tested. This has been found with inflammasomes and will likely be a conserved phenomenon. Whether sensors are strictly intracellular, or cell surface receptors are also involved, is also currently unknown. 

The complexity of tissue- and cell-specific responses, in relation to PANoptosis, should also be considered and further investigated. PANoptosis is a fluid process due to the many ways cell death can be executed. Some routes of cell death may be favored in certain tissues or cell types. Additionally, the PANoptotic response may be executed differently in various cell types based on differences in gene expression of key cell death molecules that exist among cells. It is also worth noting that many cancers are largely associated with dysregulation of apoptosis, further highlighting pathway specificity in some contexts. Understanding the subtle nuances which make each tissue and cell type unique may be useful in determining how PANoptosis occurs in order to harness this process for therapeutic purposes. 

Furthermore, there is a need to mechanistically understand the regulation of PANoptosis, as this could be the key to developing new clinical therapeutics for human cancers and diseases. IRF1 has been implicated as a central upstream regulator [2,4,8,75], and other components of this pathway should be elucidated. Inhibiting certain molecular components of cell death may enhance the activation of others, as has been observed previously [15,137], and this requires careful consideration during the drug design process. Perhaps targeting one aspect of cell death will result in subpar clinical therapies due to the redundancy of PANoptotic molecules. 

PANoptosis has been implicated across the disease spectrum, including in cerebral ischemia [114]; bacterial, viral, and fungal infections [3,9,10,11,12,13,14,15], including oral infections [115,116]; inflammatory diseases [2,5,16,18,20]; cancers [4,7,8]; and cancer therapies [4,7,117,118]. It will be important to improve our understanding of this pathway and identify how previous descriptions of crosstalk, plasticity, redundancies, molecular switches, and interconnectedness among cell death processes fit within this inclusive concept to gain a holistic picture of cell death. Only when we identify all the ingredients in the PAN can we begin to effectively target these molecules and develop novel therapeutics to save lives and improve patient outcomes. 

## Figures and Tables

**Table 1:** Totality of cell death in cellular responses.

Trigger	PANoptosome Sensor	Regulator	Pyroptosis		Apoptosis		Necroptosis		PANoptosis	
			Genotype	Cell Death?	Genotype	Cell Death?	Genotype	Cell Death?	Genotype	Cell Death?
**IAV** [9,10,11,133]	ZBP1	IRF1	*Nlrp3* ^−/−^	**✔**	*Casp8* ^DA/DA^	**✔**	*Ripk3* ^−/−^	**D**	*Fadd* ^−/−^ *Ripk3* ^−/−^	**D**
			*Casp1/11* ^−/−^	**✔**	*Casp6* ^−/−^	**D**	*Mlkl* ^−/−^	**✔**	*Casp8* ^−/−^ *Ripk3* ^−/−^	**X**
							*Ripk1* ^K45A^	**✔**	*Casp1/11* ^−/−^ *Casp8* ^−/−^ *Ripk3* ^−/−^	**X**
**KPT + IFN** [7]	ZBP1	IRF1	*Nlrp3* ^−/−^	**✔**	*Casp3* ^−/−^	**✔**	*Ripk3* ^−/−^	**D**	*Casp8* ^−/−^ *Ripk3* ^−/−^	**X **
			*Asc* ^−/−^	**✔**						
			*Casp1* ^−/−^	**✔**	*Casp7* ^−/−^	**✔**	*Mlkl* ^−/−^	**✔**		
			*Casp11* ^−/−^	**✔**						
***Francisella*** [12,134]	AIM2	IRF1	*Aim2* ^−/−^	**X**			*Ripk3* ^−/−^	**D**	*Casp8* ^−/−^ *Ripk3* ^−/−^	**X**
			*Casp1/11* ^−/−^	**X**						
			*Asc* ^−/−^	**X**						
			*Mefv* ^−/−^	**D**					*Fadd* ^−/−^ *Ripk3* ^−/−^	**X**
			*Nlrp3* ^−/−^	**✔**						
			*Nlrc4* ^−/−^	**✔**						
**HSV1** [12]	AIM2		*Aim2* ^−/−^	**X**			*Ripk3* ^−/−^	**D**	*Casp8* ^−/−^ *Ripk3* ^−/−^	**X**
			*Mefv* ^−/−^	**D**						
			*Nlrp3* ^−/−^	**✔**						
			*Nlrc4* ^−/−^	**✔**						
***Yersinia*** [3]	RIPK1		*Casp1/11* ^−/−^	**✔**	*Casp3* ^−/−^	**✔**	*Ripk3* ^−/−^	**✔**	*Casp8* ^−/−^ *Ripk3* ^−/−^	**D**
							*Ripk1* ^−/−^	**D**		
			*Gsdmd* ^−/−^	**✔**	*Casp7* ^−/−^	**✔**	*Mlkl* ^−/−^	**✔**	*Casp1/11* ^−/−^ *Casp8* ^−/−^ *Ripk3* ^−/−^	**X**
**TAK1i** [5,20]	RIPK1		*Nlrp3* ^−/−^	**✔**			*Ripk3* ^−/−^	**✔**	*Casp8* ^−/−^ *Ripk3* ^−/−^	**X**
			*Aim2* ^−/−^	**✔**			*Ripk1* ^−/−^	**X**		
			*Nlrc4* ^−/−^	**✔**			*Ripk1* ^K45A^	**X**		
			*Asc* ^−/−^	**✔**						
**TNF + IFN-γ** [8]		IRF1	*Casp1/11* ^−/−^	**✔**	*Casp3* ^−/−^	**D**	*Ripk3* ^−/−^	**✔**	*Casp8* ^−/−^ *Ripk3* ^−/−^	**X**
			*Casp1* ^−/−^	**✔**						
			*Casp11* ^−/−^	**✔**	*Casp7* ^−/−^	**✔**			*Fadd* ^−/−^ *Ripk3* ^−/−^	**X**
**MHV** [15]			*Nlrp3* ^−/−^	**I**			*Ripk3* ^−/−^	**D**	*Casp8* ^−/−^ *Ripk3* ^−/−^	**X**
			*Casp1/11* ^−/−^	**I**						
			*Aim2* ^−/−^	**✔**						
			*Nlrc4* ^−/−^	**✔**					*Casp1/11* ^−/−^ *Casp8* ^−/−^ *Ripk3* ^−/−^	**X**
			*Casp11* ^−/−^	**✔**						
***Salmonella*** [10,30,135,136]			*Nlrc4* ^−/−^	**D**			*Ripk3* ^−/−^	**✔**	*Casp8* ^−/−^ *Ripk3* ^−/−^	**✔**
			*Casp1/11* ^−/−^	**D**						
			*Casp11* ^−/−^	**D**					*Casp1/11* ^−/−^ *Casp8* ^−/−^ *Ripk3* ^−/−^	**X**
			*Asc* ^−/−^	**D**						
***Pseudomonas*** [137,138,139]			*Nlrc4* ^−/−^	**D**					*Casp1/11* ^−/−^ *Casp8* ^−/−^ *Ripk3* ^−/−^	**X**
			*Asc* ^−/−^	**✔**						
			*Casp1* ^−/−^	**X**						

Studies have consistently identified cell death crosstalk, plasticity, redundancy, interconnection, and molecular switches in evaluations of disease and cellular phenotypes. The totality of biological effects in these studies cannot be individually accounted for by pyroptosis, apoptosis, or necroptosis alone, leading to the conceptualization of PANoptosis. This table focuses on cell death in murine bone marrow-derived macrophages as a model. For genotypes representing a disruption in each programmed cell death pathway, the presence or absence of cell death (Cell death?) is denoted for each. **✔**, cell death occurs at levels similar to those seen in wild-type cells; **D**, decreased cell death as compared with wild-type; **I**, increased cell death as compared with wild-type; **X**, no cell death.

**Table 2:** Totality of cell death in disease phenotypes.

Model	Pathology	Pyroptosis		Apoptosis		Necroptosis		PANoptosis	
		Genotype	Disease?	Genotype	Disease?	Genotype	Disease?	Genotype	Disease?
***Pstpip2*^cmo^** [16,18,140,141]	Osteomyelitis	*Nlrp3* ^−/−^	**✔**	*Casp8* ^−/−^ *Ripk3* ^−/− a^	**✔**	*Ripk3* ^−/−^	**✔**	*Nlrp3* ^−/−^ *Casp8* ^−/−^ *Ripk3* ^−/−^	**X**
		*Casp1* ^−/−^	**✔**					*Casp1* ^−/−^ *Casp8* ^−/−^ *Ripk3* ^−/−^	**X**
***Sharpin*^cpdm^** [142,143,144,145,146]	Dermatitis	*Nlrp3* ^−/−^	**D**	*Bid* ^−/−^	**D**	*Mlkl* ^−/−^	**D**	*Ripk3* ^−/−^ *Fadd* ^E-KO^	**X**
						*Ripk3* ^−/−^	**D**	*Ripk3* ^−/−^ *Tradd* ^E-KO^	**X**
		*Casp1/11* ^−/−^	**D**			*Ripk1* ^K45A^	**X**	*Casp8* ^−/−^ *Ripk3* ^−/−^	**X ^b^**
***Ptpn6*^spin^** [147,148]	Dermatosis	*Nlrp3* ^−/−^	**✔**	*Casp8* ^−/−^ *Ripk3* ^−/− a^	**✔**	*Ripk3* ^−/−^	**✔**	*Casp8* ^−/−^ *Ripk1* ^−/−^ *Ripk3* ^−/−^	**X **
						*Mlkl* ^−/−^	**✔**		
		*Casp1* ^−/−^	**✔**			*Ripk1* ^K45A^	**✔**		
***Hoil*deficiency** [149]	Embryonic lethality			*Casp8* ^−/−^	**✔**	*Ripk3* ^−/−^	**D**	*Casp8* ^−/−^ *Mlkl* ^−/−^	**D**
				*Casp8* ^−/−^ *Ripk3* ^−/− a^	**D** ^c^	*Mlkl* ^−/−^	**D**	*Casp8* ^−/−^ Ripk1^−/−^ *Ripk3* ^−/−^	**X**
						*Ripk1* ^K45A^	**D**		
**Caspase-8 deficiency** [62,81,82,83,84,131]	Embryonic lethality			*Casp8* ^−/−^	**✔**	*Ripk1^D^* *^138^* *^N^*	**D**	*Casp8* ^−/−^ *Ripk3* ^−/−^	**X**
								*Casp8* ^−/−^ *Mlkl* ^−/−^	**X**
***Casp8*^C362A^** [130]	Embryonic lethality	*Nlrp3* ^−/−^ *Mlkl* ^−/−^	**✔**	*Fadd* ^−/−^ *Mlkl* ^−/−^	**D**	*Mlkl* ^−/−^	**✔**	*Casp1/11* ^−/−^ *Ripk3* ^−/−^	**X**
		*Casp1* ^−/−^ *Mlkl* ^−/−^	**✔**						
		*Casp11* ^−/−^ *Mlkl* ^−/−^	**D**			*Ripk3* ^−/−^	**D**	*Casp1/11* ^−/−^ *Mlkl* ^−/−^	**X**
		*Asc* ^−/−^ *Mlkl* ^−/−^	**D**						
**LPS shock** [2,150]	Lethality	*Asc* ^−/−^	**✔**			*Ripk3* ^−/−^	**✔**	*Casp8* ^−/−^ *Ripk3* ^−/−^	**X**
								*Casp8* ^−/−^ *Ripk3* ^K51A^	**X**
		*Casp11* ^−/−^	**D**					*Casp8* ^−/−^ *Ripk3* ^−/−^ *Ripk1* ^−/−^	**X**
**TNF + IFN-γshock** [2]	Lethality					*Ripk3* ^−/−^	**✔**	*Casp8* ^−/−^ *Ripk3* ^−/−^	**X **
**FlaTox injection** [151,152]	Hypothermia	*Nlc4* ^−/−^	**X**	*Casp8* ^−/−^ *Ripk3* ^−/− a^	**✔**	*Ripk3* ^−/−^	**✔**	*Casp1* ^−/−^ *Ripk3* ^−/−^	**D**
		*Asc* ^−/−^	**✔**					*Casp1* ^−/−^ *Casp8* ^−/−^ *Ripk3* ^−/−^	**X**
		*Casp1* ^−/−^	**D**					*Asc* ^−/−^ *Casp8* ^−/−^ *Ripk3* ^−/−^	**X**
		*Casp1/11* ^−/−^	**D**						
		*Gsdmd* ^−/−^	**D**						

^a^*Casp8*^−/−^*Ripk3*^−/−^ genotype is considered as an apoptosis deletion only when the phenotype is the same as the *Ripk3*^−/−^ genotype, showing that the added deletion of apoptotic caspase-8 does not affect the phenotype. ^b^ *Sharpin*^cpdm^*Casp8*^−/−^*Ripk3*^−/−^ mice could be born but did not survive to weaning. ^c^ *Hoil*^−/−^*Casp8*^−/−^*Ripk3*^−/−^ mice succumb at late gestation through a process that appears to be independent of cell death, while *Hoil*^−/−^*Casp8*^+/−^*Ripk3*^−/−^ mice undergo cell death-induced loss of yolk sac vascularization to contribute to lethality. Disease phenotypes in mouse models are associated with many different cell death molecules. Deletion of specific combinations can alleviate disease. The totality of biological effects in these studies cannot be individually accounted for by pyroptosis, apoptosis, or necroptosis alone, leading to the conceptualization of PANoptosis. For genotypes representing a disruption in each programmed cell death pathway, the presence or absence of disease (Disease?) is denoted for each. **✔**, disease or lethality occurs at levels similar to those seen in wild-type animals; **D**, decreased disease or lethality as compared with wild-type; **X**, no disease or lethality (rescued).

## Data Availability

Not applicable.
